# Probucol Pharmacological and Bio-Nanotechnological Effects on Surgically Transplanted Graft Due to Powerful Anti-Inflammatory, Anti-Fibrotic and Potential Bile Acid Modulatory Actions

**DOI:** 10.3390/pharmaceutics13081304

**Published:** 2021-08-20

**Authors:** Armin Mooranian, Corina Mihaela Ionescu, Susbin Raj Wagle, Bozica Kovacevic, Daniel Walker, Melissa Jones, Jacqueline Chester, Thomas Foster, Edan Johnston, Momir Mikov, Marcus D. Atlas, Hani Al-Salami

**Affiliations:** 1 The Biotechnology and Drug Development Research Laboratory, Curtin Medical School & Curtin Health Innovation Research Institute, Curtin University, Bentley, Perth, WA 6102, Australia; A.Mooranian@curtin.edu.au (A.M.); c.ionescu@postgrad.curtin.edu.au (C.M.I.); susbinraj.wagle@postgrad.curtin.edu.au (S.R.W.); bozica.kovacevic@postgrad.curtin.edu.au (B.K.); danieljcswalker@gmail.com (D.W.); melissa.a.jones@postgrad.curtin.edu.au (M.J.); j.chester@student.curtin.edu.au (J.C.); thomas.p.foster@student.curtin.edu.au (T.F.); edan.johnston@student.curtin.edu.au (E.J.); 2 Hearing Therapeutics, Ear Science Institute Australia, Queen Elizabeth II Medical Centre, Nedlands, Perth, WA 6009, Australia; marcus.atlas@earscience.org.au; 3 Department of Pharmacology, Toxicology and Clinical Pharmacology, Faculty of Medicine, University of Novi Sad, Hajduk Veljkova 3, 21101 Novi Sad, Serbia; momir.mikov@mf.uns.ac.rs

**Keywords:** probucol, transplantation, pancreatic beta-cells, interleukin-17, interleukin-12, surgery, bile acid profile

## Abstract

Introduction. A major obstacle in islet transplantation and graft survival pre and post transplantation is islet apoptosis due to mainly inflammatory bio molecules released during islet harvesting and post graft transplantation and hence, subsequent graft fibrosis and failure. This study aimed to investigate if incorporation of the anti-inflammatory anti-hyperlipidaemic drug probucol (PB) would improve islet-graft survival and function, post transplantation in Type 1 diabetes (T1D). Methods. T1D was induced in mice, and biological profiles of the diabetic mice transplanted PB-microencapsulated islets harvested from healthy syngeneic mice were measured. Results and Conclusion. Compared with sham (no PB), the treated group showed significant reduction in serum levels of interleukin-1β, interleukin-6, interleukin-12, interleukin-17, and tumour necrosis factor-α, accompanied by a 3-fold increase in survival duration, which suggests PB islet-protective effects, post transplantation.

## 1. Introduction

Diabetes is a debilitating metabolic condition, which is divided into two main types, Type 1 diabetes (T1D) and Type 2 diabetes (T2D). Diabetes complications include cardiovascular diseases, eye and nerve damage and renal failure due to mainly poor maintenance of glucose homeostasis and diabetes-associated inflammation [1]. Drug treatments for diabetes vary in types and dosages, depending on the type and severity of diabetes. Insulin therapy was discovered in 1921 and remains a mainstream therapy for people with T1D, and more than a third of people with T2D. Despite strict adherence to insulin therapy, the majority of diabetic patients have inadequate control of glycemia and suffer from diabetes-associated complications [2]. Notwithstanding its significant benefits in treating diabetes, insulin therapy has many limitations, including stringent storage requirements, route of administration being an injection or a pump, which compromises patients’ compliance, and variable and fluctuating dosing requirements. Other compounds have been trialled for their potential hypoglycaemic effects, but to date, insulin injections remain the only mainstream treatment for T1D. Transplantation of viable and functional islets of Langerhans to diabetic patients has been proposed as a new way to supplement or even replace insulin therapy. Nevertheless, and in spite of extensive and ongoing research in islet transplantation, it remains a challenge and its wide applications in the clinic remain somewhat elusive and unestablished. The main challenges to islet transplantation include host inflammation and subsequent fibrotic growth and graft failure. Published studies have suggested that host inflammation to transplanted islets can be overcome by islet encapsulation prior to transplantation. Islet encapsulation can be achieved by using the artificial cell microencapsulation (ACM) technology [3,4,5,6,7,8,9,10,11,12]. 

ACM technology for therapeutic delivery and transplantation was pioneered and developed by Thomas Chang at McGill University (Canada) in the 1960s and 1970s, and since then, this technology has been widely studied by many laboratories, scientists, researchers and clinical and translational entrepreneurs. In islet encapsulation, the technology relies on the use of chemical polymers to encapsulate viable islets of Langerhans, providing physical supporting 3D-structure, while maintaining porous scaffolds and microcapsules that facilitate exchange of nutrients and wastes with the outside biological environment, post transplantation, and hence, ensure islet survival and an ability to function as a mini pancreas. The size of the pores of the scaffolds and microcapsules should be large enough to allow and facilitate permeation of needed nutrients, waste materials and insulin, but small enough to prevent immune cell permeating through and attacking encapsulated islets [5,6,7,8,9,10,11,12,13,14,15,16]. For islet encapsulation, many polymers have been trialled using the ACM technology, including the seaweed-based polymer, alginate. 

Alginate has shown promise as the main encapsulating polymer in islet transplantation; however, inflammation and fibrotic growth post alginate-encapsulation of islets and graft transplantation remain major challenges to long-term islet delivery and success in T1D treatment [17,18]. Recent studies have shown that host inflammation has been found to encompass high levels of oxidants, free radicals, and proinflammatory biomolecules such as interleukin-1b (IL-1β), interleukin-6 (IL-6), interleukin-12 (IL-12), interleukin-17 (IL-17), interferon-gama (IFN-γ), and tumour necrosis factor-alpha (TNF-α) [15,16,17,19,20]. One way to optimise alginate-based islet transplantation is via islet co-encapsulation with powerful anti-inflammatory drugs capable of reducing secretion of proinflammatory biomolecules and subsequent fibrotic growth, and optimise the chance of graft success, particularly in the long term. Ideally, the co-encapsulated drug is well established and has demonstrated strong and powerful anti-inflammatory effects. It would be advantageous if this drug also has direct cellular protective effects, particularly on pancreatic β-cell and has shown to optimise β-cell viability, performance, and insulin secretion. An example of such a drug is probucol (PB). 

PB is an anti-hypercholesteremic drug with several unique and powerful pharmacological and biological effects, including antioxidant, antifree radicals and anti-inflammatory effects [21]. In addition, when co-encapsulated with a mouse-cloned cell line of pancreatic β-cells, PB exerted anti-inflammatory effects as well as direct desirable cellular protective effects, enhancing cellular viability, performance, and insulin secretion [22]. Other studies have examined molecular mechanisms underpinning the anti-inflammatory effects of PB. Recent studies have demonstrated PB’s potency in specifically targeting NF-κB cellular pathways, suppressing stimulation and recruitment of neutrophils, and attenuating secretion of proinflammatory biomarkers [21,23]. Additionally, PB has been used to improve recovery following spinal cord injury due to its effects in reducing inflammatory cytokines [23]. Owing to its antioxidant and anti-inflammatory effects, PB has also been examined in other inflammatory disorders such as heart failure [24], atherogenesis [25], neurovascular inflammation [26], and diabetes [22], demonstrating significant desirable effects. Interestingly, recent studies have shown a strong association between diabetes development and the bile acid profile, particularly reduced levels of secondary bile acids [27], and hence, the bile acid profile may contribute to transplantation outcome in T1D. Thus, PB co-encapsulation with islets pre transplantation may result in optimised islet viability, performance and glycaemic control, reduced inflammation, and improved transplantation outcome in T1D, potentially via modulation of the bile acid profile. 

Accordingly, this study aimed to investigate if PB co-encapsulation with microencapsulated islets will form stable microcapsules capable of controlling hyperglycaemia, reduce inflammation and influence the bile acid profile, in T1D mice.

## 2. Materials and Methods

All experiments were approved by the Animal Ethics Committee at Curtin University (protocol JARE2017-7, approved in July 2017), and all experiments were performed according to the Australian Code of Practice for the care and use of animals for scientific purposes.

### 2.1. Formulation and Islet-Microcapsule Formation

PB, the bile acids chenodeoxycholic acid (CDCA), lithocholic acid (LCA) and ursodeoxycholic acid (UDCA), and alloxan powder (>97%) were purchased from Sigma-Aldrich (St. Louis, MO, USA) and Thermo Fisher (Melbourne, VIC, Australia). Transplantation tools were purchased from ABLE Scientific (Melbourne, VIC, Australia). Materials used for islet microencapsulation were also purchased from Thermo Fisher (Melbourne, VIC, Australia), Scharlab Ltd. (Melbourne, VIC, Australia), and Sigma-Aldrich (St. Louis, MO, USA). Sodium alginate (low viscosity), poly-l-ornithine, barium chloride, and water-soluble ultrasonic gel were purchased from Sigma-Aldrich, and Scharlab Ltd. Prepared solutions consisted of 40 mM barium chloride (bathing gel), 1.2% sodium alginate, 0.8% poly-l-ornithine, 1% water-soluble ultrasonic gel, and 2.5% PB. The mixtures were prepared by either dissolving ingredients in water or simply using the manufacturers’ purchased liquid products and make that up to the final mixture with water using stirring and heating as described below. Deionised water was used as the main vehicle, and mixtures were stirred for 2–3 h at 37 °C followed by 6–7 h at room temperature, and heated to 37 °C 30 min prior to use. Mixtures that were not used were stored in the refrigerator or −20 °C freezer, and prior to use, they were placed at room temperature for 6–7 h and heated at 37 °C for 30 min prior to use. If mixtures were not stored in the refrigerator or at −2 °C (i.e., stored at room temperature), they were used within 24 h of preparation, unless stored at −80 °C in the freezer, in which case they were used within 1 month of preparation. Regardless of the storage temperature deployed, all mixtures were stirred for at least 30 min and up to 180 min, at 37 °C, prior to use. Remaining mixtures and solutions were stored for up to one month, at −80 °C in the freezer, post preparation, except for alloxan, which was used immediately after preparation. Once formulations were ready for microcapsule formation and islet transplantation, a new set-up of aseptical techniques were deployed. The overall processes of islet encapsulation and microcapsule formation took less than 60 min and were done aseptically. Preparation of formulations and islet encapsulation were carried out aseptically in customised fume hoods, using our well-established and approved protocols [14,15,28]. For cell confocal imaging, cellular staining of pancreatic β-cell line, NIT-1 cells, grown in vitro, was carried out using specialised staining agents as appropriate, sourced and supported by the Curtin Health Innovation Research Institute, Bentley, Australia, as described in our published methods [6,16,20]. When microcapsules were not being analysed, they were kept in the refrigerator for up to a month before being discarded. When microcapsules were required for imaging, size analyses or surface composition measurements, they were taken out of the refrigerator and dried to allow such analyses, as per our established methods and as appropriate [10,19,29]. In brief, after microcapsules formation, they were stored within the bathing gel for up to 21 days in the refrigerator. In order to dry the microcapsules, they were taken out of the refrigerator, placed at room temperature for 8 h, followed by being placed at a controlled-hot room for 48 h (at 37 °C and relative humidity of 35%). Relative humidity was set as the weight of water vapour for each unit of weight of air, and was expressed as grams of water vapour per each kilogram of air, using the Angelantoni Environmental and Climatic Test Chamber (Italy), as per our established-methods [30,31]. Imaging, size analysis, and surface composition measurements were carried out simultaneously at multiple research centres including: the Curtin University Electron Microscope Facility at Bentley, the John De Laeter Research Centre and the Pharmaceutical Technology Laboratory, at Curtin University, as well as the CISRO research centre in Perth, Western Australia, using our established methods [10,29]. 

### 2.2. Processes of Islet Extraction and Transplantation (Balb/c Mice)

**Figure 1:**
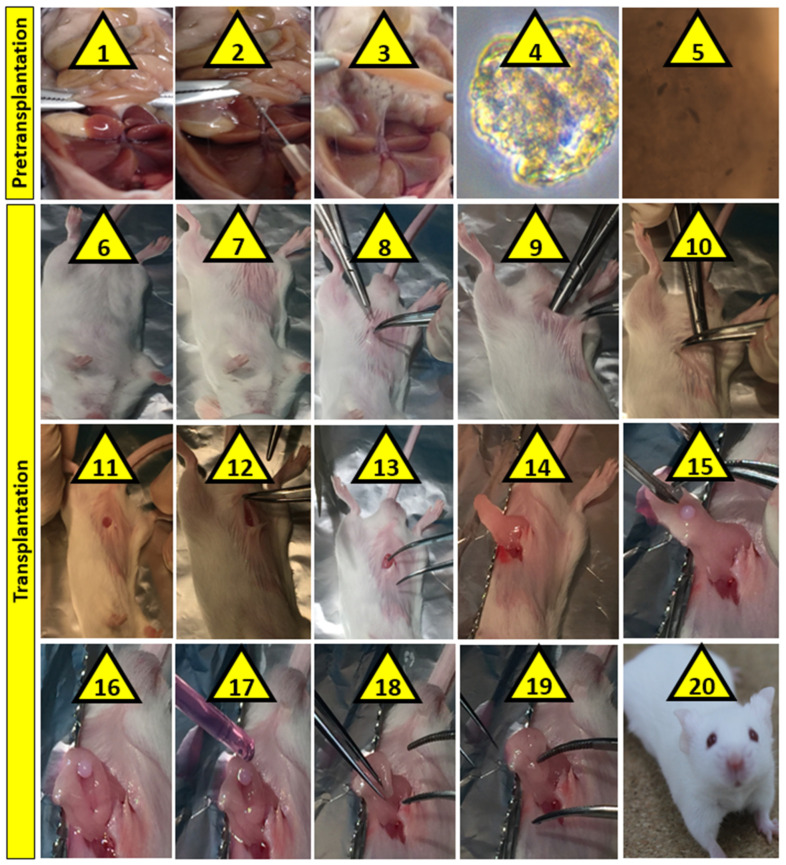
An illustration surgical figure: (1) location of the common bile duct, (2) injection of collagenase into the common bile duct, (3) perfusion and separation of the pancreas, (4) extracted and purified islets, (5) PB-impregnated microencapsulated islets used for transplantation, (6–14) creation of an omentum pouch for transplantation, and (15–20) transplantation and mouse recovery.

Once mixtures and formulations were prepared, islets were harvested, digested, separated, rejuvenated in fresh media, and encapsulated in formulations to produce microcapsules, via our Ionic Gelation Vibrational Jet Flow technology [10]. For islet extraction and preparation, male 6–8-week-old balb/c mice were acquired from the Animal Resources Centre (Perth, Australia) and islets harvested using procedures that have been approved by the Animal Ethics Committee at Curtin University and the Animal Resource Centre (Perth, Australia). In order to extract islets from the donor mice, the abdomen of the euthanised donor mouse was opened, and collagenase enzyme solution was injected to the bile duct (at a concentration of 1 mg/mL in Roswell Park Memorial Institute media, RPMI), meticulously and carefully to avoid any unintended tissue damage. The extracted pancreatic islets were subjected to gentle swirling and digestion for 19 min at 37 °C. The digestion was ceased by using RPMI media supplemented with 10% fetal bovine serum. The pancreatic tissues were purified by filtering through micrometer-wide wire mesh and repeated centrifugation at 2500 rpm for several min each time, on slow centrifugation mode, in order to avoid islet damage and reduction in viability. Prior to microencapsulation, the extracted islets were rejuvenated by being cultured in RPMI media supplemented with 10% FBS, 5.5 mM glucose and 100 µg/mL penicillin streptomycin for best filtrate. Once encapsulated, islets were then surgically transplanted into both groups of the diabetic mice, control (no PB) and treatment (with PB). Surgical transplantation was carried out, as per our approved ethics application, supported by the Curtin Animal Ethics Committee, at Curtin University, Bentley (Perth), Western Australia, Australia. Microencapsulated islets were transplanted within 2 h of being encapsulated, while the microcapsules were kept in RPMI media until transplantation. For transplantation surgical illustration, freshly prepared islet-microcapsules were prepared and transplanted into freshly euthanised mice (Figure 1). All procedures of islets extraction and transplantation were carried out in accordance with the relevant regulations as well as the technical and husbandry standard operating procedure in the Animal Resources Centre and in the Life Sciences Research Facility at Curtin University. All animal experiments were performed according to the Australian Code of Practice for the care and use of animals for scientific purposes.

### 2.3. Imaging Analyses

For the light microscopy imaging of the microcapsules, YS2-H optical, Nikon (Tokyo, Japan) was used, while for the scanning electron microscopy, and confocal imaging as well as the surface composition measurements Zeiss Neon 40EsB FIBSEM (Tescan, Brno, Czech Republic), UltraVIEW Vox, Perkin Elmer (Waltham, MA, USA), and Oxford Instruments, Aztec X-Act (Abingdon, U.K) were used as per our well-established procedures [32,33,34]. In brief, for the YS2-H optical imaging, dry microcapsules were placed on a glass slide and multiple images taken at different angles. The best image with best resolution was selected and presented. For the Zeiss Neon 40EsB FIBSEM scanning electron microscopic imaging, microcapsules were dried then coated with platinum, and using laser-guided pen, multiple images were taken. The best images with clear morphology relevant to specific desired magnifications were presented. For the UltraVIEW Vox confocal imaging complemented and equipped with a Yokogawa CSU-X1 confocal scanning unit, microcapsules with stained cells were imaged and multiple images taken, with the best resolution being presented.

### 2.4. Diabetes Induction and Blood Glucose and Insulin Measurements

Alloxan powder was mixed with saline and injected subcutaneously to mice at a dose of >150mg/kg body weight to induce diabetes. In order to ensure the most accurate alloxan dose per mouse, each mouse was weighed immediately prior to alloxan injection. Blood glucose measurements were carried out prior to mice injection with alloxan to establish a healthy baseline, then measurements were taken daily thereafter. Once blood glucose concentration was >13 mM in two consecutive days, insulin concentrations were measured to confirm diabetes. All mice became diabetic within two to three days after a single injection of alloxan. AccuCheck Glucose meter was used Roche, North Ryde NSW, Australia) for glucose measurements, while Mercodia ultrasensitive insulin kits (Mercodia, Uppsala, Sweden) were used for insulin analysis. For glucose and insulin measurements, either tail vein or saphenous vein blood was used. Daily glucose measurements as well as insulin confirmation for diabetes induction were performed on all mice, and required less than 30 µL of blood samples for each measurement. T1D was confirmed by glucose and insulin measurements as well as mice exhibiting classic symptoms of the disease, such as urination and weight loss. In our severe and well-established T1D mouse model [27], mice exhibited severe symptoms of diabetes and showed no detectable insulin levels, demonstrating severe stage of T1D with blood glucose rising sharply and reaching >16 mM for all mice, by the second day of the experiment. Once T1D was confirmed, both groups of mice (control and treatment) were transplanted with the microcapsules. Urination and weight loss were some of the visible symptoms the diabetic mice exhibited, and no insulin was administered during the experiment, based on the approved animal ethics application and as per study design. When mice reached end points of weight loss and urination, they were euthanised as per approved protocols. Weight loss was accepted within 10% and all mice lost between 7 and 10% after diabetes induction. Our model of T1D is robust and all mice exhibited clear signs of the disease, where control mice had to be euthanised within six days of alloxan injection. Despite islet transplantation, control mice did not show improved glycaemic control and continued to deteriorate in terms of diabetes symptoms and well-being, and hence, were euthanised once end points were reached as per approved protocols and in order to ensure that best animal welfare practices were strictly followed. 

### 2.5. Study Design and Quantification of Cytokines and the Bile Acid Profile

**Figure 2:**
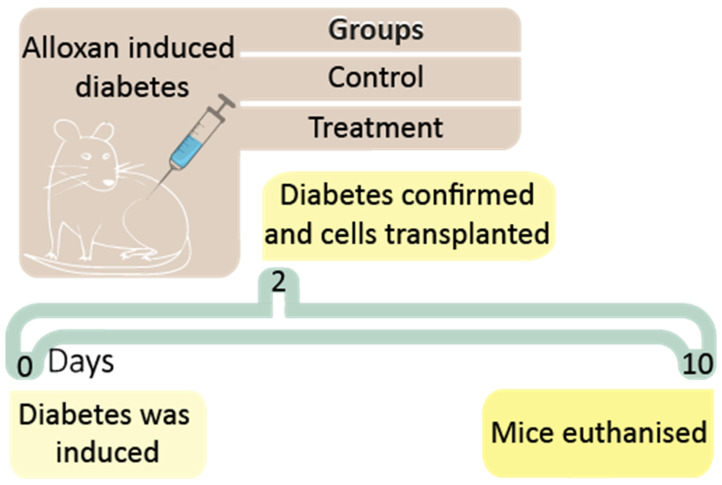
Timeline for the in vivo study to examine the therapeutic effect of transplanted PB-islet microcapsules.

Adult male balb/c mice were randomly divided into two equal groups (6–7 per group), one control and one treatment. All mice were acclimatised for several days prior to the experiment. Using alloxan, T1D was induced in all mice, and mice became diabetic within two days of alloxan administration. Islets from donor mice (adult male balb/c mice) were harvested and encapsulated ready for transplantation. Upon T1D confirmation in Day = 2, all animals were transplanted either sham (microencapsulated islets without PB) or treatment (microencapsulated islets with PB). Each mouse received 80 viable islets. At Day = 10, mice were euthanised, and blood, liver, and faeces collected for cytokine and bile acid analyses (Figure 2). 

Blood, liver, and faecal samples were collected, prepared, and analysed for plasma concentrations of IL-1β, IL-6, IL-12, IL-17, IFN-γ, and TNF-α, as well as plasma, liver, and faecal concentrations of the bile acids, CDCA, LCA and UDCA. 

For analyses of IL-1β, IL-6, IL-12, IL-17, IFN-γ, and TNF-α in plasma, cytokine bead array (CBA) kits were acquired from BD Biosciences (San Jose, CA, USA) and were used as per the manufacturer’s instructions and as per our well-established methods [35,36,37]. For each sample, sample processing and mixing involved protein extraction, solubilisation, reagent dilution, and precipitation, and the concentrations of the cytokines were determined and analysed by Attune Acoustic Focusing flow cytometry (Carlsbad Life Technologies, Carlsbad, CA, USA) using refined methods described in depth in our previously published papers [34,35,36]. 

For analysis of the bile acids CDCA, LCA, and UDCA in plasma, liver, and faeces, liquid chromatography mass spectrometry (LCMS) was used, based on our well-established methods [19,27,38]. Briefly, 30 mg of samples or plasma were mixed with ice acetonitrile in a one-to-one ratio to extract the three bile acids, and 40 µL of the bile acid-containing samples were diluted twofold in mobile phase. The LCMS system deployed was a Shimadzu LCMS 2020 (Shimadzu Corporation, Kyoto, Japan). The LCMS system flow rate was established at 0.25 mL/min for each run of the mobile phase at ambient temperature and pH adjusted to 3. The column used was Phenomenex C18 with pore size of 5 µm, length of 10 cm, and internal diameter of 2 mm. The column was washed and pre-equilibrated with the mobile phase for each run. Ten µL of pooled and prepared samples were injected into the detector by the injector within the SIL 20AC HT Shimadzu-Prominence autosampler (Shimadzu Corporation, Kyoto, Japan). Blank samples contained only mobile phase, while quality control samples contained known concentrations of the bile acids in the analysed medium. Quality control samples at high, medium, and low concentrations were prepared. Quality control concentrations covered the range from 5 to 800 ng/mL, and were prepared by spiking plasma, liver, or faecal samples with quality control solutions of known concentrations of the three bile acids. Quality control and blank samples were included in each analysis. The quality control samples were prepared independently of calibration standards but in the exact same way and were used to ensure method robustness, i.e., for batch-level quality control as well as method reproducibility and stability. The limit of quantification and limit of detection were obtained and verified based on the signal-to-noise ratios of 3 and 10, respectively. The bile acid analyses and concentrations measurements were based on our well-established protocols [19]. 

## 3. Results and Discussion

**Figure 3:**
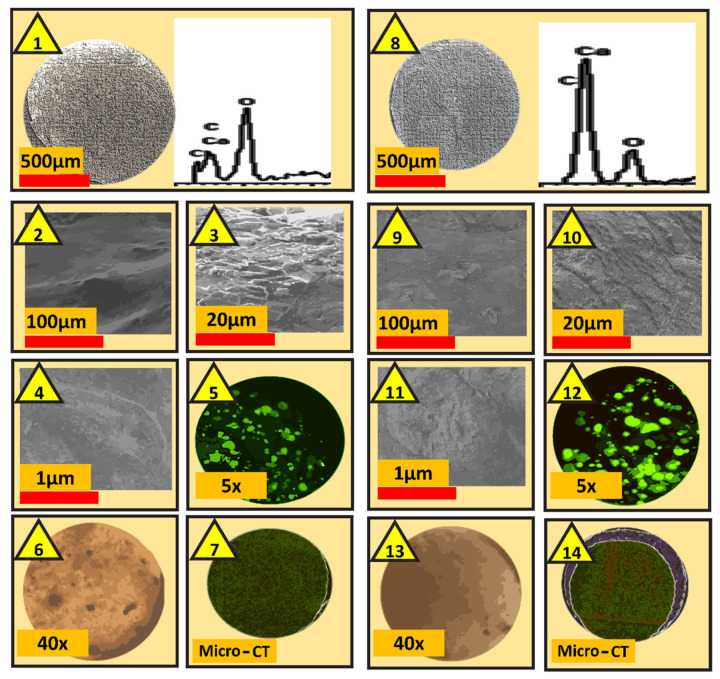
Imaging and surface composition of control (no PB; 1–7) and treatment (with PB; 8–14) microcapsules. Images 1–4 and 8–11 are SEM and EDXR, while images 5 and 12 are confocal. Images 6 and 13 are optical while images 7 and 14 are Micro-CT.

**Figure 4:**
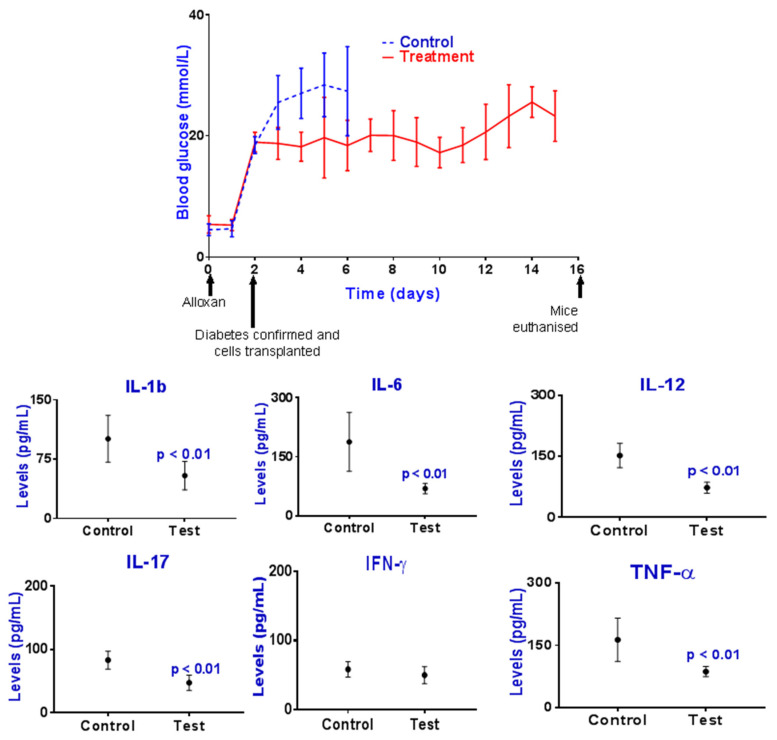
Postprandial blood glucose and plasma cytokines concentrations for control and test groups. Data are mean ± SEM.

**Figure 5:**
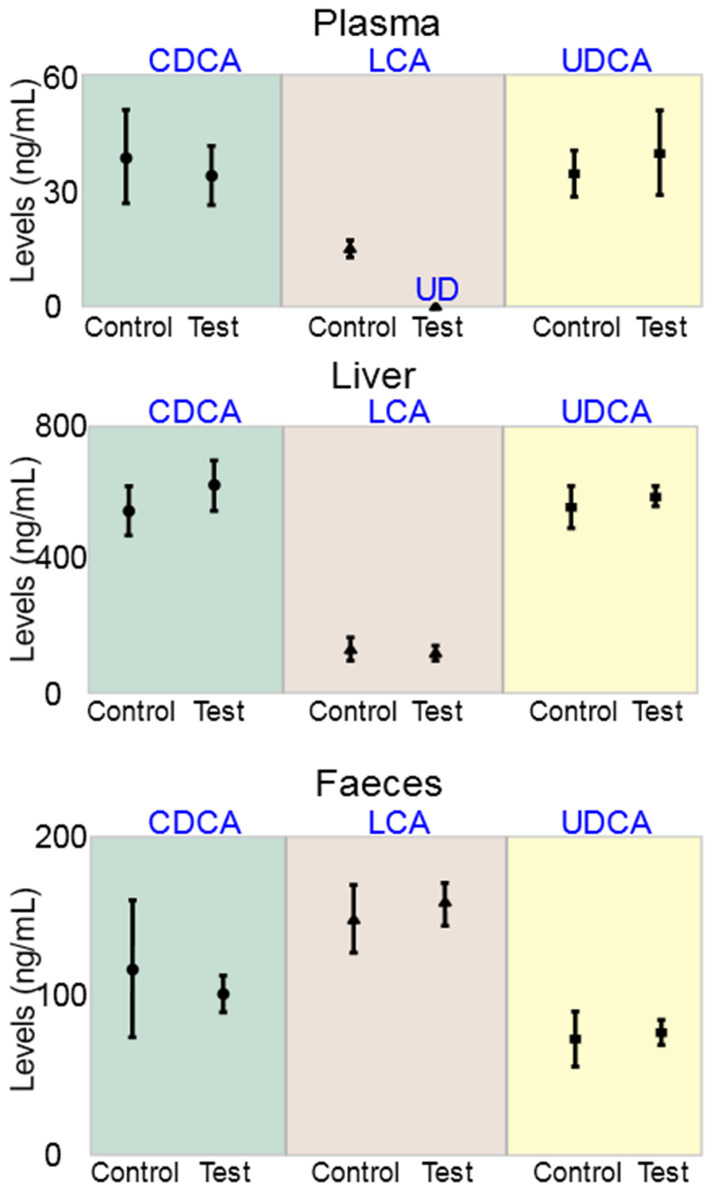
Bile acid concentrations in samples collected from the plasma, liver and faeces.

Imaging, topographic, and surface analysis results are presented in Figure 3, while postprandial blood glucose concentrations and plasma concentrations of the biomarkers IL-1β, IL-6, IL-12, IL-17, IFN-γ, and TNF-α are presented in Figure 4. The concentrations of the bile acids CDCA, LCA, and UDCA in plasma, liver, and faeces are presented in Figure 5. 

### 3.1. Imaging, Topography, and Surface Analysis Measurements

Microcapsules’ imaging in Figure 3 represented randomly selected microcapsules from several produced batches. During imaging, various scales were used such as 500 µm, 100 µm, 20 µm, and 1 µm magnifications. The imaging techniques deployed (SEM and Micro-CT) showed opaque, discrete, consistent, and spherical shaped microcapsules, and cell and islet (confocal and optical) images showed presence of cells and islets within all layers of the microcapsules, which suggests efficient and consistent method of encapsulation regardless of PB presence. The images also suggest that PB distribution within the microcapsules did not compromise the microcapsules’ shape, size, uniformity, surface features, topography, or layers’ thickness or cell and islet distribution within the microcapsules. In addition, the surface elemental composition analysis (EDXR) showed that no PB-specific atoms such as S were present on the surface of the microcapsules, which suggests that PB incorporation was efficient and remained within the core of the microcapsules rather than distributed throughout the layers of the microcapsules including the surface. The results are consistent with previous studies in our lab that showed that excipient co-encapsulation within alginate-based microcapsules did not alter the morphological and topographic features of the microcapsules [29]. However, PB entrapment within the core of the microcapsules seems to be dependent on the encapsulating methodology, the design of the microcapsules, and type and ratio of encapsulating excipients. In a recent study in our laboratory, PB capsules designed for oral targeted delivery exhibited PB distribution throughout the layers of the microcapsules including the surface, suggesting variation of PB distribution depending on techniques deployed and the nature of the excipients [39]. The accumulation of PB within the core of the microcapsules suggests that PB molecules interact directly with the encapsulated viable islets and would likely exert some biological and anti-inflammatory effects on the islets and perhaps on the treated mice (Figure 4). 

### 3.2. Blood Glucose and Inflammatory Cytokines Measurements

The treatment group showed lower concentrations of daily blood glucose compared with control, and mice survived longer, suggesting that PB incorporation with the encapsulated islets resulted in improved islet-response to hyperglycaemia, insulin secretion, and glycaemic control. The improved glycaemic control also suggests that the nature of the microcapsules and encapsulating materials, as well as the internal 3D architecture including the microcapsule’s membrane, were suitable and supportive of protein and gas exchange and insulin diffusion into the outside biological fluids, resulting in insulin release from the microcapsules and subsequent hypoglycaemic effects. The inflammatory profile was also improved in the treatment group compared with control. The treatment group showed lower concentrations of plasma IL-1β, IL-6, IL-12, IL-17, and TNF-α. The anti-inflammatory effects associated with PB incorporation suggest powerful and direct inhibition of immune cell secretion of these biomarkers, possibly via islet-triggered positive effects. Similar to the hypoglycaemic effects of PB incorporation, the anti-inflammatory effects seem to result from PB core-distribution within the microcapsules and direct interaction with encapsulated islets, as shown in Figure 3. The anti-inflammatory effects of PB may have resulted from improved glycaemic control or reduced islets’ own inflammatory profile, resulting in reduction in systemic inflammation and ameliorated secretion for these biomarkers. Such PB anti-inflammatory effects are less likely to be a direct result from PB presence in the transplanted graft and its diffusion to the systemic circulation and directly affecting the immune cells, particularly since the amount of incorporated PB within the microcapsules was not large enough to warrant such powerful and systemic anti-inflammatory effects. When compared with published studies, the observed anti-inflammatory effects of PB on viable islets are in line with the literature. In a recent study in our laboratory, when PB was incorporated with a mouse-cloned viable pancreatic β-cell, within microcapsules, PB increased cellular viability, insulin release and significantly reduced the proinflammatory biomarker TNF-α, in vitro [39]. In another study where PB was orally administered to mice induced with T1D, PB microcapsules brought about a significant reduction in the inflammatory profile, which suggests that PB exerted significant and powerful anti-inflammatory effects in T1D [40]. Similarly, in a study published by Shimizu H. et al., the authors investigated the application of PB in diabetic mice administered multiple low-dose streptozotocin injections. The aim of the study was to investigate the effects of PB on the protection against pancreatic β-cell damage from oxidative stress caused by streptozotocin. The authors concluded that PB incorporation resulted in improved insulin release, attenuated hyperglycemia, and improved glycaemic control post oral glucose challenge test, hence, protecting pancreatic β-cell from oxidative stress [41]. Another study carried out by Guoyong Hu et al. showed similar positive effects of PB in a hamster animal model of hypertriglyceridemic acute pancreatitis and strong anti-inflammatory effects on pancreatic islets [42]. Accordingly, our findings of PB effects on islets are in line with the literature and support PB positive effects on transplanted islets and subsequent improvement in glycaemic control and inflammation. Such PB effects may have been associated with observed alteration in the bile acid profile due to diabetes induction and glycaemic control (Figure 5).

### 3.3. Bile Acid Measurements

The concentrations of the bile acids CDCA, LCA and UDCA in control and treatment mice were similar in liver and faeces. The concentrations of LCA in plasma were undetected in the treatment group compared with control, showing significant reduction of LCA plasma concentrations as a result of PB incorporation in the encapsulated and transplanted islets. PB effects on LCA were only observed in plasma and not in liver or faeces, which may be associated with reduced inflammation or improved glycaemic control, in the treatment group compared with control. Based on the literature, reduced inflammation seems to be the likely cause of reduced LCA concentrations in plasma, in the treatment group. In a recent study in our laboratory which compared the bile acids profiles between healthy and T1D mice, LCA concentrations were significantly increased in the T1D mice compared with healthy mice, not only in plasma, but also in liver and faeces [29], while in another study in insulin-resistant mice, plasma LCA concentrations were higher compared with healthy [19], and in both studies there was an increase in inflammation in the diabetic animals compared with the healthy animals. However, significant and well-defined association between high LCA concentrations in plasma and tissue, and inflammation has not been demonstrated by other researchers. In fact, some studies have demonstrated beneficial and anti-inflammatory effects of LCA. In a study by Moretti A. et al., the authors investigated the effects of nanotherapeutics containing LCA-based macromolecules on inflammation and potential applications in atherosclerosis. The authors showed that LCA-conjugated macromolecules reduced inflammation in macrophages via protein synthesis pathways [43]. In another study by Ward J. et al. the authors investigated the applications of the bile acids LCA and UDCA in inflammatory bowel diseases. The authors showed that both bile acids exerted anti-inflammatory effects in the colon and concluded that LCA is a potent inhibitor of intestinal inflammation, and hence may have potential applications in inflammatory bowel diseases [44]. Overall, the reduction in LCA plasma concentrations presented in Figure 5 is likely to be influenced by multiple factors including cholesterol catabolism, gut microbiome, the bile acid enterohepatic recirculation in gut and liver, and diabetes-associated inflammation and hyperglycaemia. However, exact mechanisms of LCA change remain elusive.

## 4. Summary

In summary, our study investigated if PB pre-encapsulation with primary viable islets will improve islets functions, post encapsulation and transplantation in T1D. The physical features of the PB graft, its antidiabetic and anti-inflammatory effects, as well as its effects on the bile acid profile, were assessed. PB graft showed improved glycaemic effects, anti-inflammatory effects and LCA-altering effects, suggesting potential applications of PB in islet transplantation, with potential mechanisms of actions including effects on the expression and synthesis of well-known inflammatory biomarkers, cholesterol metabolism and bile acid profile, and cell survival and functions.

## Data Availability

The data are available on request from the corresponding author.
